# Virulence and Draft Genome Sequence Overview of Multiple Strains of the Swine Pathogen *Haemophilus parasuis*

**DOI:** 10.1371/journal.pone.0103787

**Published:** 2014-08-19

**Authors:** Susan L. Brockmeier, Karen B. Register, Joanna S. Kuehn, Tracy L. Nicholson, Crystal L. Loving, Darrell O. Bayles, Sarah M. Shore, Gregory J. Phillips

**Affiliations:** 1 Virus and Prion Diseases Research Unit, National Animal Disease Center, Agricultural Research Service, USDA, Ames, Iowa, United States of America; 2 Ruminant Diseases and Immunology Research Unit, National Animal Disease Center, Agricultural Research Service, USDA, Ames, Iowa, United States of America; 3 Department of Veterinary Microbiology and Preventive Medicine, College of Veterinary Medicine, Iowa State University, Ames, Iowa, United States of America; 4 Infectious Bacterial Diseases Research Unit, National Animal Disease Center, Agricultural Research Service, USDA, Ames, Iowa, United States of America; Quuen's University Belfast, United Kingdom

## Abstract

*Haemophilus parasuis* is the cause of Glässer's disease in swine, which is characterized by systemic infection resulting in polyserositis, meningitis, and arthritis. Investigation of this animal disease is complicated by the enormous differences in the severity of disease caused by *H. parasuis* strains, ranging from lethal systemic disease to subclinical carriage. To identify differences in genotype that could account for virulence phenotypes, we established the virulence of, and performed whole genome sequence analysis on, 11 *H. parasuis* strains. Virulence was assessed by evaluating morbidity and mortality following intranasal challenge of Caesarean-derived, colostrum-deprived (CDCD) pigs. Genomic DNA from strains Nagasaki (serotype 5), 12939 (serotype 1), SW140 (serotype 2), 29755 (serotype 5), MN-H (serotype 13), 84-15995 (serotype 15), SW114 (serotype 3), H465 (serotype 11), D74 (serotype 9), and 174 (serotype 7) was used to generate Illumina paired-end libraries for genomic sequencing and *de novo* assembly. *H. parasuis* strains Nagasaki, 12939, SH0165 (serotype 5), SW140, 29755, and MN-H exhibited a high level of virulence. Despite minor differences in expression of disease among these groups, all pigs challenged with these strains developed clinical signs consistent with Glässer's disease between 1–7 days post-challenge. *H. parasuis* strains 84-15995 and SW114 were moderately virulent, in that approximately half of the pigs infected with each developed Glässer's disease. *H. parasuis* strains H465, D74, and 174 were minimally virulent or avirulent in the CDCD pig model. Comparative genomic analysis among strains identified several noteworthy differences in coding regions. These coding regions include predicted outer membrane, metabolism, and pilin or adhesin related genes, some of which likely contributed to the differences in virulence and systemic disease observed following challenge. These data will be useful for identifying *H. parasuis* virulence factors and vaccine targets.

## Introduction

*Haemophilus parasuis* is a Gram negative, fastidious bacterium requiring a source of nicotinamide adenine dinucleotide (NAD) for growth that belongs to the family *Pasteurellaceae*. Members of this family range from important pathogens to commensals of animal and human mucosa. *H. parasuis* infects pigs, and like the members of this family in general, strains appear to range from non-pathogenic isolates that are carried in the upper respiratory tract, to isolates that invade and cause a bacteremia with subsequent lesions characterized by various combinations of meningitis, polyserositis (inflammation of serous membranes), and polyarthritis, often referred to as Glässer's disease. Although probably often overlooked, this bacterium can also contribute to animal disease by causing rhinitis, otitis media, and pneumonia [1]–[5].

*H. parasuis* infection causes significant loss to the swine industry each year throughout the world. Disease occurs most often in nursery age through early finishing swine (from about 4 weeks to 4 months of age) and is often associated with stressful events, such as weaning or moving to the finishing barn [6]. This also corresponds to times of comingling when transmission of new strains and coinfections often occur. The outcome of infection depends on both the bacterial genetic and phenotypic characteristics and the host's immune status. Transmission from dam to piglet occurs early, often under the protection of passive maternal immunity, with subsequent development of an active immune response [7], [8]. Because of this, experimental reproduction of disease with pathogenic strains can be inconsistent in conventionally reared animals, whereas colostrum-deprived pigs provide a reproducible model of disease [9], [10].

Fifteen serotypes of *H. parasuis* have been identified, although it is not unusual for strains to be non-typeable with the typing sera currently available [11]. Several serotypes may circulate simultaneously within a herd, but certain serotypes, including serotypes 4 and 5, appear to be isolated most frequently from cases of Glässer's disease [12], [13]. Heterologous protection, especially with the use of bacterins, appears to be imperfect and may depend on the serotypes involved and the virulence of the strains [14], [15]. Cross protection has been demonstrated after natural infection with avirulent strains or even vaccination with attenuated members of the Pasteurellaceae family [16], [17]. Virulence factors for *H. parasuis* are not well defined. The capsule is believed to play a role in virulence by increasing the resistance to complement-mediated killing and phagocytosis by macrophages [18], [19]. Other factors that have been associated with virulence include porin proteins, cytolethal distending toxin, and trimeric autotransporters (VtaA) [20]–[23].

Although *Haemophilus influenzae* was the first bacterium to have its genome sequenced in 1995, genomic information is only now beginning to accumulate for *H. parasuis*. The first genome sequence of *H. parasuis* made publically available was a draft sequence of strain 29755 (GenBank accession no. ABKM00000000) in 2008, followed by the complete genome sequence of SH0165 [24], published in 2009. Determining the genome sequences of multiple strains of *H. parasuis* displaying varied and defined virulence phenotypes should contribute to a better understanding of this microorganism. This approach is confounded, however, since there is insufficient information confirming the pathogenicity or virulence of different strains in direct comparisons. For some strains, virulence has been established on the basis of experimental reproduction of disease, but the pathogenicity of most strains is assumed on the basis of clinical signs observed in the pig of origin [25]. Isolates from pigs with clinical signs and lesions consistent with Glässer's disease are presumed to be virulent, while those obtained from the upper respiratory tract of healthy pigs are generally presumed to be avirulent. Unfortunately, this is an imprecise measure of virulence since conditions such as coinfection could enhance the apparent virulence of some strains and existing immunity may mask the true virulence potential of others. In this report, we address these deficiencies by establishing the virulence of 10 genetically distinct *H. parasuis* isolates in Caesarean-derived, colostrum deprived (CDCD) pigs, a reference model free from many confounding factors inherent in alternative model systems. We sequenced the genomes of these isolates to generate a body of data useful for unlocking genetic links to disease outcome for this important swine pathogen and provide some early insights based on initial comparative analysis.

## Materials and Methods

### *H. parasuis* strains

Details of the strains of *H. parasuis* included in this study (29755, 12939, MN-H, SW114, 174, H465, D74, SW140, 84-15995, Nagasaki, and SH0165) are given in Table 1.

**Table 1:** *H. parasuis* strains.

Strain	Serotype	Origin	Isolation Site	Diagnosis	MLST Type	Refs.
**Nagasaki**	5*	Japan	Meninges	Septicemia/meningitis	24	[11], [12], [26]–[30]
**12939**	1,4	US-IA	Lung	Pneumonia/polyserositis	115	[16]
**SH0165**	5	China	Lung	Polyserositis	31	[24], [17], [31]
**SW140**	2*	Japan	Nose	Healthy	28	[11], [12], [30]
**29755**	5	US-IA	Lung	Polyserositis	8	[9], [10], [16], [32]
**MN-H (19851)**	13	US-MN	Unknown	Polyserositis	116	[16]
**84-15995**	15*	US-SD	Lung	Pneumonia	15	[11], [12]
**SW114**	3*	Japan	Nose	Healthy	26	[11]–[12], [16], [30], [33]
**H465**	11*	Germany	Trachea	Pneumonia	20	[12]
**D74**	9*	Sweden	Unknown	Unknown	25	[11], [12]
**174**	7*	Switzerland	Nose	Healthy	4	[11], [12], [30]

*Serotype Type Strain.

### *H. parasuis* culture

*H. parasuis* strains and samples collected from pigs were cultured on Casman's agar base (BD BBL, Franklin Lakes, NJ) (CAS) supplemented with 5% filtered horse serum (GIBCO, Life Technologies, Grand Island, NY) and 0.01% (w/v) nicotinamide adenine dinucleotide (NAD) (Sigma-Aldrich, St. Louis, MO) at 37°C in 5% CO_2_ for 24 hours (inocula), 48 hours (pig samples), or overnight (genomic DNA isolation). A culture suspension with an A_600_ of 0.42 was prepared in phosphate buffered saline (PBS), which yields a titer of approximately 10^8^ CFU/ml, and pigs were given 1 ml (0.5 ml/nostril) of this inoculum intranasally. PBS was used for sham inoculum. Multilocus sequence typing (MLST) was performed on strains prior to inoculation and from *H. parasuis* reisolated from at least 1 pig in each experimental group to verify strain identity [34]. MLST profiles were also used to verify the correct assignment of genome sequences to each of the isolates.

### Swine virulence experiments

Two separate experiments were completed for the virulence studies. To derive the CDCD piglets, mixed breed sows from a high health status herd that is antibody negative for PRRSV and influenza were purchased. Caesarian sections were performed and the piglets were placed in individual isolation cages for the first week and then group housed in isolation rooms. Each experimental group consisting of a different *H. parasuis* strain was housed in a separate isolation room. Nasal swabs were taken prior to starting the experiment and plated on sheep bood agar and Casman's media to look for the presence of *H. parasuis* and other common swine bacterial pathogens. In the first experiment, eighty, 9-week-old, CDCD pigs were randomly assigned to 8 experimental groups of 10 pigs each. Pigs were intranasally inoculated with one of 7 strains of *H. parasuis* (12939, SW140, D74, 84-15995, Nagasaki, SW114, or SH0165) or sham inoculated with PBS. In the second experiment, 33 CDCD pigs were randomly assigned to 5 experimental groups and inoculated with 1 of 4 strains of *H. parasuis* [29755 (8 pigs), MN-H (7 pigs), 174 (7 pigs) or H465 (7 pigs)] or sham inoculated with PBS (4 pigs). Nasal swabs were collected on day 0 and on day 3 post-challenge for culture of *H. parasuis*. Rectal temperatures were taken daily beginning 2 days before challenge through day 7 after challenge; pigs with a rectal temperature greater than 40°C were considered febrile. Clinical signs were recorded daily for the duration of the experiment. Whole blood samples were taken on days 3 and 5 post-challenge in the first experiment and days 1, 3 and 5 post-challenge in the second experiment and 100 µl was plated on CAS agar plates for *H. parasuis*. Pigs that died or were euthanized due to presence of clinical signs were necropsied and individual swabs of pericardium, thoracic cavity, abdominal cavity, meninges, and joints were collected and placed in 1 ml of PBS and 100 µl of this as well as serum and lung lavage collected at necropsy were cultured for *H. parasuis*. At necropsy gross lesions were recorded for all pigs.

*Ethics statement:* Animal studies were conducted in accordance with the recommendations in the Guide for the Care and Use of Laboratory Animals of the National Institutes of Health. The animal experiments were approved by the USDA-National Animal Disease Center's Institutional Animal Care and Use Committee (protocol #2436). During the course of the experiment pigs were examined for clinical signs approximately every 4 hours except for an 8 hour overnight period and any pig showing signs of severe Glässer's disease, such as neurologic involvement and/or severe lameness or depression that resulted in recumbency with reluctance to stand, were euthanized.

### Genomic DNA Isolation and Sequencing

Genomic DNA was isolated from overnight cultures using a Wizard Genomic DNA purification kit (Promega, Madison, WI) and quantified with PicoGreen (Molecular Probes, Eugene, OR). Sequencing libraries were prepared using the Illumina Paired-End Sample Preparation Kit according to the manufacturer's protocol but with the recommended modification of performing gel extraction incubations at room temperature [35]. Prior to sequencing, library quality and insert size were evaluated on an Agilent Bioanalyzer 2100 DNA 7500 chip. Libraries were sequenced at the Iowa State University DNA Facility on an Illumina GAIIx for either 75 cycles (strains SW114, 12939, 84-15995, H465, and D74) or 100 cycles (strains Nagasaki, MN-H, 29755, 174 and SW140). GenBank accession numbers for the genomes are given in Table 2 and Kuehn et al. [36].

**Table 2:** *H. parasuis* genome sequences general summary.

Strain	SH0165	Nagasaki	SW114	MN-H	12939	29755	84-15995	H465	D74	174	SW140
**Status**	Finished	Draft	Draft	Draft	Draft	Draft	Draft	Draft	Draft	Draft	Draft
**Accession No.**	CP001321	APBT*	APBU*	APBV*	APBW*	ABKM*	APBX*	APBY*	APBZ*	APCA*	APCB*
**Size (bp)**	2,269,156	2,178,597	1,938,544	1,761,086	1,953,029	1,512,980	2,090,833	1,498,510	2,229,567	1,369,811	1,413,419
**GC (%)**	40.0%	39.9%	39.7%	39.7%	39.8%	39.7%	39.8%	39.7%	39.6%	39.9%	39.6%
**ORF count**	2,021	2,308	1,947	1,859	1,974	1,616	2,206	1,540	2,218	1,433	1,484
**Total tRNA**	56	51	34	34	40	35	38	42	43	19	27
**Total rRNA**	20	8	5	8	6	6	5	10	7	5	6
**5S rRNA**	8	5	3	5	4	2	3	8	5	3	3
**16S rRNA**	6	3	1	2	1	2	1	2	1	1	1
**23S rRNA**	6	1	1	1	1	2	1	1	1	1	1

SH0165 data from Xu *et al.*2011.

*Accession No. = four letters listed followed by 00000000.

### Genome Sequence Assembly, Annotation and Analysis

Sequence quality filtering, format conversion and assembly were carried out using NextGENe software v2.00 (Softgenetics, State College, PA). Briefly, Illumina fastq files were quality filtered and trimmed to exclude reads with less than 25 bases, a median quality score below 20, ≥3 bases with quality scores ≤20, and more than 3 uncalled bases. Contigs were assembled using SH0165 as a reference scaffold. For each assembly, at least one cycle of the NextGENe condensation-elongation tool was applied using forward-reverse read balancing. DeBruijn based, paired-end assembly for each strain was carried out using estimated library sizes based on respective Agilent Bioanalyzer 2100 readings and overlapping contiguous sequences (contigs) were merged.

Contigs were provided to the Institute for Genome Sciences (IGS) Annotation Engine at the University of Maryland School of Medicine in Baltimore, Maryland for annotation. Sequences were ordered based on homology to the *H. parasuis* SH0165 genome and merged into pseudomolecules, which were then annotated using the IGS Manatee annotation and analysis tool. The availability of the annotated draft sequences and the corresponding NCBI accession numbers have been previously reported [36].

The mGenomeSubtractor tool [37] was used with BLASTP default settings to compare the predicted protein sequences of all the isolates sequenced here with selected genes of *H. parasuis* SH0165 (NC_011852).

The nucleotide sequences aligned for capsular loci comparisons of *H. parasuis* strains were obtained from joined contig arrangements encompassing the following regions [Strain, position (loci)]: SH0165 48,840–67,151 (HAPS_0038-0055), Nagasaki 5,767–23,566 (HPSNAG_0006-0022), SW114 147,836–167,968 (HPSSW114_1194-1213), MN-H 830–7,403(HPSMNH_0002-0020), 12939 150,260–173,574 (HPS12939_0666-0689), 29755 527–18,690 (HPS_0043-0061), 84-15995 2,507–22,024 (HPS8415995_0003-0024), H465 10,765–34,537 (HPSH465_0008-0030), D74 2,510–22,143 (HPSD74_0329-0350), 174 4,753–27,939 (HPS174_0013-0037) and SW140 211,660–236,003 (HPSSW140_0610-0634). The entirety of the capsule locus was found to be present on a single contig within each draft whole genome assembly for each isolate except MN-H. The Illumina MN-H assembly was gapped at two locations within the locus, which were resolved as follows. A 57.6 Kb contig containing the complete capsule locus of MN-H was identified in a previously obtained Roche 454 assembly. Alignment with the assembled Illumina data confirmed the presence of only two sequencing gaps, with apparent lengths of 273 bp and 3156 bp; the alignment was otherwise identical throughout the locus. The Roche 454-derived MN-H capsule locus sequence was used as a bait sequence for retrieving all Illumina reads, including those not previously assembled in the prior assembly of Illumina data. The reads that mapped to the capsule locus were *de novo* assembled using MIRA v. 3.9.15 [38]. This provided an ungapped capsule locus sequence for MN-H identical to the sequence obtained using the Roche 454 data (GenBank Acc. #KF841370), indicating there were no pyrosequencing homopolymer miscalls in the capsule locus assembly.

Multiple genome alignments were performed using the Mauve (v2.3.1) progressiveMauve alignment tool with default settings adjusted as necessary to visualize differences within highly similar regions [39], [40]. Other multiple sequence alignments were performed using the ClustalW2 v2.0 multiple sequence alignment server or MUSCLE (v3.8) through the European Bioinformatics Institute (EMBL-EBI) Web Services [41]–[43]. BLASTP with default settings was used to compare the amino acid sequences of predicted CDS regions within and flanking predicted capsular regions of all strains against each other.

In addition, whole genome sequences of the strains Nagasaki and D74 were analyzed using the RAST Server [44]. Annotated putative protein sequences were mapped to functional gene categories or subsystems in RAST for functional comparison and BLASTP was used to compare putative protein sequences between the strains. A graphical representation of the BLASTP results was generated using CGView [45].

The 30 *vta*A genes, as discussed by Pina et al., were downloaded from Genbank by their accession numbers [20]. The downloaded *vta*A genes were grouped into their three reported classes, and the sequences in each class were aligned with ClustalW2 [42]. The HMMER tools (HMMER v. 3.1b1) were used to build (hmmbuild) a Hidden Markov Model (HMM) for each of the three alignments, to concatenate the models (hmmpress), and to search (hmmsearch) for the *vta*A genes in each of the assembled genomes [46]. The *vta*A genes identified in each genome were subsequently extracted and compared by BLASTN to the database of the 30 known *vta*A genes.

The PAI Finder (https://www.gem.re.kr/paidb/pai_finder.php?m=f) on the Pathogenicity Island Database was used to search for pathogenicity islands [47]. For the predicted CDS of each sequenced genome, input files containing ≤300 ORFs were generated in FASTA format and further modified with an in-house script to conform to the stylized FASTA definition line required for input into the PAI Finder tool. The genomes were submitted to the PAI Finder for analysis.

### LOS analysis

*H. parasuis* D74 and Nagasaki were cultured on Casman's agar for approximately 24 hours. A suspension was made in PBS to A600 of ∼0.5 for each isolate. Lipoologiosaccharaide (LOS) was extracted using a microextraction protocol with proteinase K digestion as described by Apicella with minor modifications [48]. Briefly, 1.5 ml of each bacterial suspension was centrifuged at 14,000× g to pellet the bacteria. The pellet was solubilized with lysis buffer (2% SDS, 4% 2-mercaptoethanol, 10% glycerol, 1 M Tris-Cl (pH 6.8) and 0.005% bromophenol blue. The solubilized bacteria were boiled for 10 min and then cooled to room temperature. Proteinase K (25 µg) was added to each lysate and the sample incubated in a 55°C water bath overnight. The sample was heated to 100°C to inactivate the proteinase K. The treated lysates we separated by SDS polyacrylamide gel electrophoresis using 4–12% bis-tris gradient gel (Invitrogen). An *Escherichia coli* LPS was included as a positive control. The gels were stained with Pro-Q Emerald 300 Lipopolysaccharide Gel Stain Kit according to manufacturer's recommendations (Molecular Probes).

## Results and Discussion

### Swine virulence experiments

**Figure 1:**
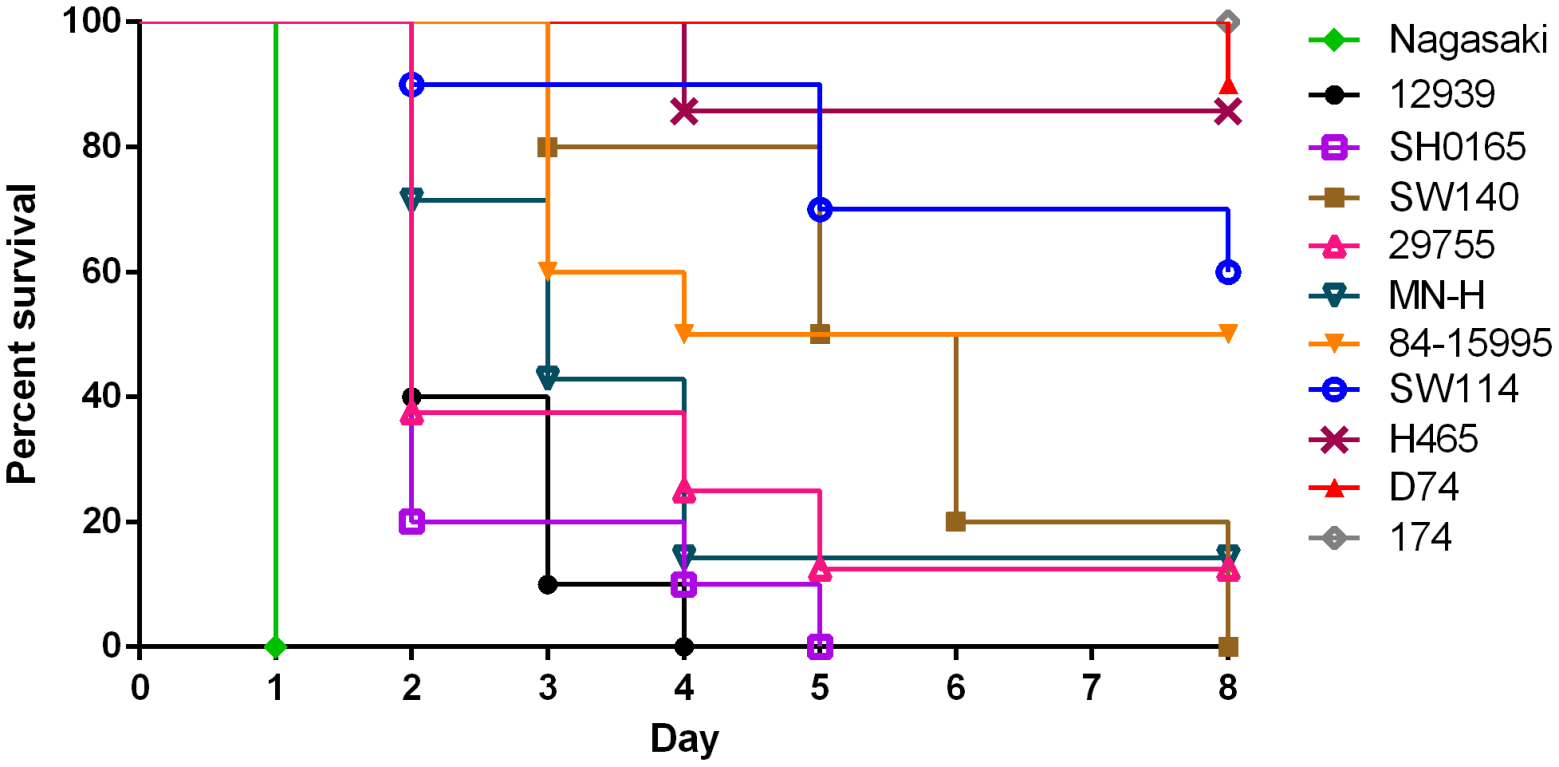
Survival rates of pigs intranasally challenged with 11 different strains of *Haemophilus parasuis*. Number of pigs infected was 10 for strains 12939, SW140, D74, 84-15995, Nagasaki, SW114, and SH0165, 8 for strain 29755, and 7 for strains MN-H, 174, and H465.

No *H. parasuis* was isolated from any of the pre-inoculation nasal swabs. Similarly, the sham-inoculated pigs demonstrated no sign of disease and no *H. parasuis* was isolated from any of these pigs at any time. The titers of the inocula, in cfu/ml, were 10^8.40^ (29755), 10^8.03^ (12939), 10^8.66^ (MN-H), 10^7.42^ (SW140), 10^8.00^ (D74), 10^8.51^ (85-15995), 10^8.66^ (Nagasaki), 10^8.09^ (SW114), 10^9.41^ (174), 10^7.97^ (H465), and 10^8.62^ (SH0165). Tables 3 and 4 outline the frequency with which *H. parasuis* was cultured pre- and post-mortem from pigs. Figure 1 outlines the survival of challenged groups, and Tables 5 and 6 respectively summarize the frequency of clinical signs and gross lesions in infected pigs. The MLST of the bacteria in each of the challenge inocula agreed with the type reported for that strain in the *H. parasuis* MLST database, and the MLST type of the *H. parasuis* reisolated from at least one pig from each group was the same as for the strain with which the pig was challenged.

**Table 3:** Frequency of *H. parasuis* culture from nasal swab taken on day 3 or blood taken on day 1, 3, or 5 post infection.

Group	Nasal Swab Day 3	Blood Day 1	Blood Day 3	Blood Day 5
**Nagasaki**	ns^a^	nd^b^	ns	ns
**12939**	4/4^c^	nd	2/4	ns
**SH0165**	2/2	nd	1/2	ns
**SW140**	10/10	nd	2/10	5/8
**29755**	3/3	2/8	0/3	2/2
**MN-H**	5/5	1/7	0/5	0/1
**84-15995**	10/10	nd	4/10	0/5
**SW114**	9/9	nd	1/9	2/9
**H465**	7/7	0/7	0/7	0/6
**D74**	10/10	nd	0/10	0/10
**174**	7/7	0/7	0/7	0/7

a No surviving pigs on that day.

b Sample not taken on that day.

c Number of samples positive per number of pigs surviving in each group on that day.

**Table 4:** Frequency of *H. parasuis* culture at necropsy from various sites from pigs that died or were euthanized after demonstrating clinical signs consistent with Glässer's disease.

Group	Number euthanized or found dead/Number in group	Pleura	Pericardium	Peritoneum	Joint	Meninges	Serum	Lung lavage
**Nagasaki**	10/10^a^	10/10	10/10	10/10	9/10	10/10	2/2	10/10
**12939**	10/10	10/10	10/10	10/10	10/10	10/10	3/3	10/10
**SH0165**	10/10	8/10	7/10	7/10	7/10	7/10	5/6	10/10
**SW140**	10/10	9/10	6/10	8/10	10/10	8/10	7/8	10/10
**29755**	7/8	3/7	2/7	4/7	5/7	3/7	7/7	3/7
**MN-H**	6/7	4/6	3/6	4/6	3/6	3/6	5/6	4/6
**84-15995**	5/10	3/5	3/5	3/5	4/5	4/5	2/5	5/5
**SW114**	4/10	4/4	4/4	4/4	2/4	3/4	0/2	4/4
**H465**	1/7	0/1	0/1	0/1	1/1	1/1	1/1	1/1
**D74**	1/10^b^	0/1	0/1	0/1	0/1	0/1	0/1	0/1
**174**	0/7	0/0	0/0	0/0	0/0	0/0	0/0	0/0

a Number of samples positive for *H. parasuis* per number sampled.

b One pig in this group was euthanized with clinical signs from a non-*Haemophilus* related infection.

**Table 5:** Frequency of clinical signs in *H. parasuis* infected pigs.

Group	Depression	Respiratory	Neurologic	Lameness
**Nagasaki**	10/10^a^	5/10	0/10	0/10
**12939**	8/10	8/10	1/10	4/10
**SH0165**	9/10	5/10	4/10	6/10
**SW140**	6/10	7/10	7/10	7/10
**29755**	8/8	0/8	4/8	7/8
**MN-H**	5/7	2/7	3/7	4/7
**84-15995**	4/10	4/10	4/10	5/10
**SW114**	1/10	1/10	2/10	3/10
**H465**	1/7	0/7	1/7	2/7
**D74**	0/10	0/10	1/10^b^	0/10
**174**	0/7	0/7	0/7	0/7

a Number of pigs demonstrating clinical sign per number of pigs in the group.

b One pig in this group was euthanized with clinical signs from a non-*Haemophilus* related infection.

**Table 6:** Frequency of gross lesions in *H. parasuis* infected pigs that died or were euthanized after demonstrating clinical signs consistent with Glässer's disease.

Group	Pleuritis	Pericarditis	Peritonitis	Arthritis	Pneumonia	Pulmonary edema
**Nagasaki**	6/10^a^	1/10	9/10	0/10	0/10	8/10
**12939**	3/10	5/10	7/10	4/10	5/10	5/10
**SH0165**	3/10	7/10	4/10	6/10	9/10	3/10
**SW140**	0/10	0/10	4/10	5/10	3/10	1/10
**29755**	1/7	3/7	2/7	7/7	1/7	0/7
**MN-H**	3/6	1/6	1/6	4/6	2/6	0/6
**84-15995**	1/5	0/5	3/5	5/5	1/5	2/5
**SW114**	0/4	0/4	4/4	2/4	0/4	1/4
**H465**	0/1	0/1	0/1	1/1	0/1	0/1
**D74** b	0/1	0/1	0/1	0/1	0/1	0/1
**174**	0/0	0/0	0/0	0/0	0/0	0/0

a Number of pigs demonstrating lesion per number of pigs sampled.

b One pig in this group was euthanized with clinical signs from a non-*Haemophilus* related infection.

*H. parasuis* strains Nagasaki, 12939, SH0165, SW140, 29755, and MN-H were highly virulent by the intranasal route in the CDCD pig model. All pigs in each of these groups developed clinical signs consistent with Glässer's disease between 1–7 days post-challenge. Pigs in the groups infected with these strains developed anorexia, lethargy and depression; neurologic signs ranging from tremors, to head tilt and paddling; lameness on front and/or back limbs; occasional cyanosis or discoloration of the extremities or ventrum; and respiratory signs including increased respiration and labored breathing. Most pigs in these groups ran fevers between 40.5° and 41.6°C. Occasionally pigs among these groups were found dead with no premonitory signs. At necropsy, lesions of serositis (pericarditis, pleuritis, and/or peritonitis) were present in pigs from all of these groups; often this was mild with increased amber serosal fluid accumulation and minimal fibrin tags. Pigs from most groups had evidence of arthritis with swollen hock, carpal, and elbow joints that often contained cloudy fluid and thickened joint capsules. The presence of pneumonia was variable, but apparent in most groups, with consolidation affecting up to 30% of the lung cranial-ventrally, and pulmonary edema was present in pigs from most groups as well. The frequency of *H. parasuis* culture positive samples varied in these groups from approximately 50% to 100% of all samples collected, but *H. parasuis* was isolated from at least two systemic sites from nearly every pig in these groups.

Although *H. parasuis* strains Nagasaki, 12939, SH0165, SW140, 29755, and MN-H could all be considered highly virulent, some distinctions among these groups were observed. The incubation period was shortest for pigs infected with strain Nagasaki with all pigs exhibiting clinical signs by 24 hours and all succumbing to disease by 32 hours post-challenge, whereas the mean time to death was longest for SW140 and the last pig in that group succumbed on day 8 post-challenge (Figure 1). While all pigs infected with 29755 and MN-H developed signs of disease, and most succumbed between 2–5 days, there was 1 survivor in each group until final necropsy at 21 days post-challenge. The surviving pig infected with MN-H exhibited evidence of systemic infection consisting of a fever of 40.9°C and lameness on day 7 post-challenge but appeared normal by day 9. *H. parasuis* was isolated from the nasal swab taken on day 3 but not from the blood cultures taken on days 1, 3 or 5. The 29755-infected survivor had evidence of systemic infection consisting of a fever of 41.4°C on day 4 and 41.8°C on day 5, but its temperature was normal on days 6 and 7. The pig displayed lethargy and depression on days 4–6 but got up readily and no lameness was seen. *H. parasuis* was isolated from the blood sample taken on day 5. These pigs were necropsied on day 21 post-challenge and no lesions were noted nor was *H. parasuis* isolated from the pigs at this time.

Pigs infected with strains Nagasaki and 12939 had the highest *H. parasuis* recovery rates; the bacterium was also isolated from a majority of sites from the pigs infected with SH0165 and SW140 (Tables 3 and 4). *H. parasuis* isolation rates from pigs infected with stains 29755 and MN-H were more variable, in particular there was a low rate of isolation from the lung of pigs infected with 29755 (Table 4). Because of the rapidity of onset of disease in pigs infected with strain Nagasaki, there was a lower incidence of clinical signs and lesions with this group, while the other groups infected with the highly virulent strains demonstrated a wider combination of clinical signs and lesions (Tables 5 and 6). Pigs infected with MN-H were the only pigs in which large amounts of fibrin deposition occurred in those with serositis (Table 6).

*H.parasuis* strains Nagasaki, SH0165, and 29755 are all serotype 5 strains that were initially isolated from pigs with signs of Glässer's disease, and the results reported here confirm previous experimental reports in which disease occurred in swine following intranasal infection [16], [17], [26]–[29]. Strains 12939 and MN-H were also initially isolated from pigs with Glässer's disease, however coinfecting agents were also identified; porcine reproductive and respiratory syndrome virus in the case of strain 12939 and porcine circovirus type 2, bovine virus diarrhea virus, *Pasteurella multocida*, and *Arcanobacterium pyogenes* in the case of strain MN-H. Similar to previous findings, strains 12939 and MN-H caused systemic disease when given intranasally to pigs [16]. Strain SW140 was initially isolated from the nose of a healthy pig [11]. There are no reports describing the outcome of intranasal infection of pigs with SW140, but it was able to cause Glässer's disease when given intraperitoneally; however, no death was reported during the 4 day observation time in that study [11]. In the current study, SW140 was virulent following intranasal challenge; though it was 3 days following challenge before the first pig succumbed. It is possible that the initial study was terminated prior to the time in which pigs would have succumbed to infection. Regardless, previous work and the current study confirm that SW140 can cause Glässer's disease.

*H. parasuis* strains 84-15995 and SW114 were moderately virulent by the intranasal route in the CDCD pig model, in that only 5/10 and 4/10 pigs, respectively, developed Glässer's disease and the rest of the pigs in each group remained healthy with no clinical signs of disease. In general, the pigs that did become ill had a clinical disease course, lesions, and *H. parasuis* isolation rate similar to those of pigs from the groups infected with the more virulent strains (Tables 3–6, Figure 1). *H. parasuis* was isolated from the nasal swabs of healthy pigs in these groups on day 3 but not from the blood taken on days 3 or 5 post-inoculation. There were no lesions noted when the pigs were euthanized 21 days after inoculation, but *H. parasuis* was cultured from the lungs of 2/5 surviving pigs infected with 84-15995.

Strain 84-15995 was initially isolated from the lung of a pig with pneumonia [11]. Like strain SW140, this strain was demonstrated to cause Glässer's disease when given to pigs intraperitoneally [11], but there are no reports describing intranasal infection in pigs. *H. parasuis* SW114 was initially isolated from the nasal cavity of a healthy pig [11]. This strain was previously thought to be avirulent based on experiments in which no clinical signs of disease and no gross lesions were observed following intraperitoneal infection of pigs [11], [33]. Mixed results were obtained when this strain was given intranasally to pigs from an experimental herd that was free of known swine pathogens [16]. In one experiment no disease was observed in 5 pigs inoculated with this strain, but in a subsequent experiment 6/16 pigs developed swollen joints and lameness. Recently, another report indicated 1 pig out of 7 infected with strain SW114 showed systemic lesions [49]. The results from the current experiment confirm that this strain is capable of causing disease.

*H. parasuis* strains H465, D74, and 174 were minimally virulent or avirulent by the intranasal route in the CDCD pig model. Only 1 pig inoculated with *H. parasuis* strain H465 exhibited observable clinical signs of disease. This pig started running a fever (41°C) beginning on day 1 post-inoculation but did not demonstrate clinical signs until day 3 when lameness was noted. By day 4 the pig was depressed and reluctant to stand, had purple discoloration of its ears and ventrum and was euthanized. Upon necropsy the pig had lesions of arthritis and *H. parasuis* was isolated from the joint, meninges, serum, and lung lavage. No other pig developed observable clinical signs, but several pigs had rectal temperatures between 40–40.5°C for 1 or 2 days post-challenge. *H. parasuis* was isolated from the nasal swabs of these pigs on day 3 but not from the blood taken on days 1, 3 or 5 post inoculation. Strains D74 and 174 presented as avirulent in this study. With the exception of one pig discussed below, all pigs in these groups remained healthy with no signs of clinical disease. *H. parasuis* was isolated from the nasal swabs of these pigs on day 3 but not from the blood taken on days 3 or 5 (pigs challenged with D74) or days 1, 3, or 5 (pigs challenged with strain 174) post inoculation. One pig in the group infected with D74 was found recumbent with paddling of the legs on day 8 post-inoculation and was euthanized. This pig had rectal temperatures between 40.3° and 42.1°C from days 2 through 7 post-inoculation, but no other clinical signs were noted during this time. On necropsy no gross lesions were noted and no *H. parasuis* was isolated from any systemic sites. A meningeal swab obtained from this pig and used to inoculate standard blood agar yielded a pure culture of an unidentified bacterium. On the basis of its colony morphology and ability to grow in the absence of NAD, the affected pig was judged to have succumbed to a non-*Haemophilus* infection. When the rest of the D74-challenged pigs were euthanized 21 days after inoculation, there were no lesions noted and no *H. parasuis* was cultured.

Strain H465 was initially isolated from the trachea of a pig with pneumonia and the diagnosis and origin of D74 is unknown [11]. There is no record of virulence testing of these strains under experimental conditions. Strain 174 was isolated from the nasal cavity of a healthy pig [11]. The strain has been given intraperitoneally with no clinical signs of disease and no gross lesions reported during the 4 day observation time [11].

### Genome Sequencing, Assembly and Characterization

Table S1 in File S1 contains a summary of the genome assembly statistics for each isolate sequenced. The average insert size is ∼450 bp while read lengths average 74–101 bases. Depending on the isolate, contigs averaged ∼16–33 Kb, with the largest being ∼411 Kb. For all isolates, N50 values are >46 Kb and the estimated average coverage is at least 100×. Assemblies for strains H465, 174, SW140 and 29755 appear the most noticeably incomplete, based on assembly size and the number of predicted coding sequences (CDSs; Table 2). Assembly sizes and the number of predicted CDSs for the remaining isolates more closely approximate those of the finished *H. parasuis* SH0165 genome. A search of each draft sequence for homologs of 132 genes from SH0165 whose products are predicted to serve important roles in basic metabolic functions [24], [31], likely to be conserved among isolates, also provides an overview of the completeness of the drafts. The fewest number of homologs (96–109) was identified from sequences of the four isolates with the smallest assemblies (Table S2 in File S1). Homologs of 114–129 of the genes were apparent in the remaining isolates, further indicating those drafts are most likely to be nearly complete.

### Comparative Genomics of Capsule Loci

**Figure 2:**
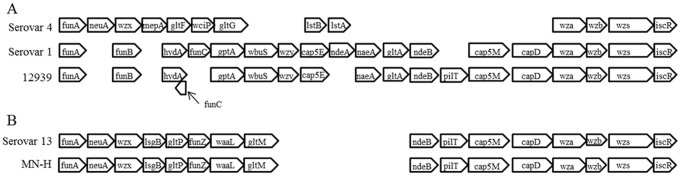
Capsule locus organization of isolates 12939 (A) and MN-H (B) compared to the capsule locus organization previously reported for the corresponding serovar reference strains [50]. Gene names are those of Howell et al [50].

Howell et al. [50] have recently reported the gene content and order of the capsule biosynthesis locus for the 15 serovar reference strains of *H. parasuis*, seven of which we sequenced for this study. Our genome sequence data corresponding to the capsule locus of those isolates (SW140, SW114, Nagasaki, 174, D74, H465 and 84-15995) are largely in agreement with theirs, although some inconsistencies were observed. They identified in SW140 and H465 (serovars 2 and 11, respectively, in addition to the serovar 1 reference strain) a CDS designated *funC* that lies between *hydA* and *gptA*, all transcribed in the same direction (see serovar 1 capsule locus organization in panel A of Figure 2). We could find no corresponding ORF in this region and noted that the *hydA* and *gptA* genes are separated by 20 bp or less. However, BLAST queries of our SW140 and H465 genome sequences with the sequence reported for *funC* (GenBank Acc.# KC795463.1) revealed the ORF to be located on the opposite strand and entirely contained within the 3′ terminus of the *hydA* gene. Another anomaly is the placement of *gltC* and *gltD* genes immediately 3′ of *funH* in the serovar 3 reference strain SW114, as indicated by the capsule locus schematic of Howell et al. [50]. The two CDSs in the corresponding location of our SW114 genome sequence are annotated as the glycosyltransferase genes *glyC* and *glyD* and their sequences are identical to GenBank entries for *glyC* and *glyD* submitted by Howell et al. [50]. We could find no GenBank submission for a *gltC* gene in *H. parasuis*, though a *gltD* gene, encoding a glutamate synthase subunit, has been identified in a number of *H. parasuis* isolates, including SW114. However, the SW114 *gltD* gene (locus tag HPSSW114_1161) lies outside the capsule locus. We conclude this anomaly may be due to mislabeling of the capsule locus schematic rather than a discrepancy in gene content.

The capsule locus sequences for 12939 and MN-H, typed as serovar 1/4 and 13, respectively, have not been previously determined. On the basis of the capsule loci annotations reported for the serovar reference isolates [50], the organization of the capsule locus from 12939 is most consistent with that of the serovar 1 reference strain (Figure 2, panel A). The presence of *pilT* and the altered location and orientation of *funC* are the only differences noted. Although 12939 was reactive with both serovar 1- and serovar 4-specific typing reagents, it lacks all the putative serovar 4-specific genes present in the variable region between *funA* and *wza*. Whether genes that lie outside the currently defined capsule locus might also affect the structure of the cell-surface polysaccharides recognized by typing sera, perhaps explaining the dual reactivity of 12939, is unknown. The inability to fully standardize serotyping reagents could also confound consistent correlations between serotyping results and capsule locus gene organization. The gene content and order found in the capsule locus of MN-H is identical to that reported for serovar 13 (Figure 2, panel B), consistent with the results of serotyping.

The location within the genome of the capsule locus for all isolates sequenced here appears to be consistent with that reported previously [50]. In all draft sequences the locus is flanked at the 5′ end by *ompP1* and genes encoding hypothetical proteins and at the 3′ end by putative iron-sulfur cluster assembly genes.

### Further Comparative Genomics

A prior study reported that the highly virulent *H. parasuis* isolates ZJ0906 and SH0165 contain pathogenicity islands with putative virulence gene homologs of the *E. coli* pathogenicity island PAI 1 and the *Salmonella* pathogenicity island SPI-1 [51]. The output from a search for pathogenicity islands in the genome sequences of the ten *H. parasuis* isolates included in this study is provided in Table 7. Neither PAI 1 nor SPI-1 was found in any isolate. No evidence was found for an exclusive association between highly virulent isolates and any particular pathogenicity island.

**Table 7:** Potential pathogenicity island homologs in *H. parasuis* isolates.

Isolate^a^	Homologous regions	Size (bp)	No. of ORFs	No. of homologs of PAI-virulence genes	PAIs homologous to this region
**Nagasaki**	1	8052	2	2	Not named (*Enterococcus faecalis* MMH594)
					Not named (*Enterococcus faecalis* V583)
**12939**	1	8203	9	2	Not named (*Enterococcus faecalis* MMH594)
					Not named (*Enterococcus faecalis* V583)
**SW140**	0				
**29755**	0				
**MN-H**	0				
**84-15995**	2	8330	9	2	Not named (*Enterococcus faecalis* MMH594)
					Not named (*Enterococcus faecalis* V583)
		5671	7	4	SCCcap1 (*Staphylococcus aureus* M)
**SW114**	1	8331	9	2	Not named (*Enterococcus faecalis* MMH594)
					Not named (*Enterococcus faecalis* V583)
**H465**	0				
**174**	3	8202	9	2	Not named (*Enterococcus faecalis* MMH594)
					Not named (*Enterococcus faecalis* V583)
		13109	14	0	GGI (*Neisseria gonorrhoeae* MS11)
		9905	8	0	YAPI (*Yersinia pseudotuberculosis* 32777)
**D74**	1	8330	9	2	Not named (*Enterococcus faecalis* MMH594)
					Not named (*Enterococcus faecalis* V583)

a listed in order of decreasing virulence.

**Figure 3:**
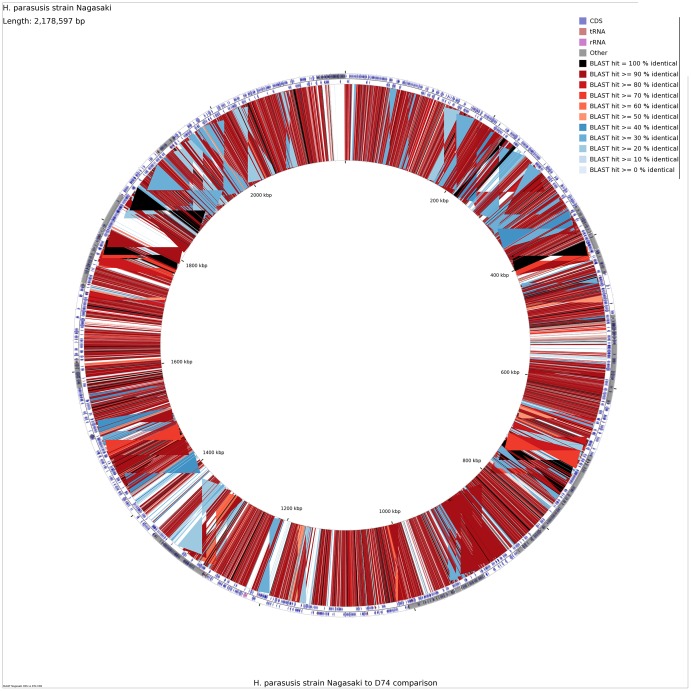
A comparison of the genetic composition of strains Nagasaki and D74 showing the result of a BLASTP analysis comparing each putative protein sequence from these strains.

To identify differences in gene content that could explain the wide range of pathotypes of the *H. parasuis* strains, sequences of the most highly virulent strain (Nagasaki) and an avirulent strain (D74) were directly compared. A comparison of the putative protein sequences from strains Nagasaki and D74 using a BLASTP analysis is shown in Figure 3. The largest difference in the genomic content observed was in phages and other mobile elements, which form the visible gaps in the alignment (Figure 3). The functional composition of the two strains did not differ greatly, with a similar distribution of genes assigned to functional categories by RAST.

**Figure 4:**
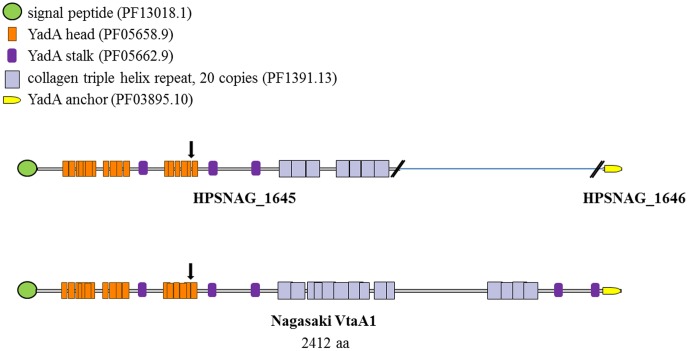
A comparison of the predicted protein structures of Nagasaki VtaA1 [20] and its highest scoring BLAST hit in the Nagasaki draft genome sequence, HPSNAG_1645. The CDS is interrupted by a break in the assembly within a region of collagen repeats. HPSNAG_1646 is a partial CDS encoding a group 1 YadA anchor domain, consistent with the group 1 anchor domain previously reported for Nagasaki VtaA1. Downward arrows indicate the location of DNA uptake signal sequences.

Recent studies provide evidence supporting a role in virulence and protection for the immunogenic proteins encoded by the *vtaA* family of trimeric autotransporter genes [20], [21], [29]. Features shared among the members of this family include YadA head domain repeats (PF05658.9), multiple YadA stalk motifs (PF05662.9), collagen triple helix repeats (PF1391.13) and a C-terminal YadA anchor region (PF03895.10). A prior analysis of eleven *H. parasuis* isolates revealed thirty *vtaA* gene variants, with only a subset of variants present in any single isolate [20]. The VtaA proteins were shown to comprise three distinct groups on the basis of the YadA anchor domain sequences. In the same study, thirteen *vtaA* genes were identified in Nagasaki, nine in group 1 and two each in groups 2 and 3. When our Nagasaki genome sequence was queried for ORFs best matching all known *vtaA* genes, eight ORFs were identified with a minimum of 97% sequence identity to at least 1.5 Kb of one or more *vtaA* genes. It was noted that six of the ORFs are incomplete, being interrupted by the terminus of a contig. Shorter regions (∼400 bp-1000 bp) of ≥98% DNA sequence identity to known *vtaA* genes were found in four additional ORFs, all interrupted by a contig break. Annotations for these CDSs are consistent with members of the *vtaA* gene family and include hep Hag family protein, collagen triple-helix repeat family protein, putative YadA-like protein and vtaA3 domain protein. Because the assembly statistics indicate the Nagasaki draft genome sequence is largely complete, it is perhaps surprising that nearly all potential *vtaA* ORFs are interrupted by contig breaks. The difficulty of properly assembling repeat sequence motifs found in the *vtaA* gene family perhaps accounts for this. In fact, at least six of the ten incomplete ORFs terminate within or immediately adjacent to the highly iterated collagen repeat regions of the predicted proteins. Also found among potential *vtaA* gene candidates were two additional incomplete ORFs, each consisting of only a YadA anchor and a short stretch of upstream sequence. Alignment of the YadA anchor domains from the nine ORFs corresponding to the C-terminal portion of a likely *vtaA* gene revealed that six represent group 1, two represent group 2 and one represents group 3, a distribution similar to that reported previously for Nagasaki [20]. While a reliable one-to-one assignment of the *vtaA*-like ORFs to specific *vtaA* genes cannot be made, best matches collectively include twelve of the thirteen *vtaA* genes previously identified in this isolate. One example of our findings is depicted in Figure 4, a comparison of the predicted domains in the previously described Nagasaki VtaA1 protein with those of its highest-scoring BLAST hit from our Nagasaki draft genome sequence (locus tag HPSNAG_1645).

In contrast, only two CDSs with a similar degree of sequence identity to *vtaA* genes could be found in the D74 draft genome, despite a level of completion equivalent to that of the Nagasaki draft genome. Both CDSs (locus tags HPSD74_0229 and HPSD74_0409) are annotated as putative YadA-like proteins and comprise complete ORFs with ∼91–95% sequence identity to roughly 1.5 Kb of the 3′ portion of known *vtaA* genes. The predicted protein structure of both CDSs includes multiple YadA head repeats and YadA stalk motifs as well as a YadA anchor, one representing group 1 and the other representing group 3. Only one additional *vtaA*-like gene was noted in the D74 genome, locus tag HPSSD74_0544. Its predicted amino acid sequence includes YadA head and YadA stalk motifs and a group 2 YadA anchor. Outside of these domains, it has no significant sequence identity to other known *vtaA* genes. The absence of collagen repeats in all three potential D74 *vtaA* family members is particularly striking. Our observations confirm and expand those of Pina et al. [20], who obtained microarray and PCR data suggesting that group 2 and group 3 *vtaA* genes may exist in noninvasive strains, albeit with highly divergent passenger domains. Though unable to identify and sequence specific *vtaA*-like genes, they found little evidence of collagen repeat sequences in the genome of noninvasive strains. Excluding the VtaA proteins of invasive *H. parasuis* strains, collagen repeats are rarely found among bacterial autotransporters. The data presented here further strengthens the argument of Pina et al. [20] that collagen repeat domains in *H. parasuis* VtaA proteins may contribute to adhesion and invasion.

Several genes encoding outer membrane proteins implicated in previous reports as potentially contributing to virulence or protection are present in both Nagasaki and D74 genome sequences and display minimal sequence heterogeneity (∼93% or greater amino acid sequence identity). These include genes for the OmlA family protein SmpA [52], PalA [53], which is highly similar to the potentially immunoprotective P6 protein of *H. influenzae*, the bacterial OB fold protein YgiW [52] and one of two loci encoding the cytolethal distending toxin [23]. The second locus encoding the toxin in D74 is truncated due to a break in the assembly, as are genes for the putative serine protease autotransporters EspP1 and EspP2, preventing a thorough comparison of the corresponding ORFs.

**Figure 5:**
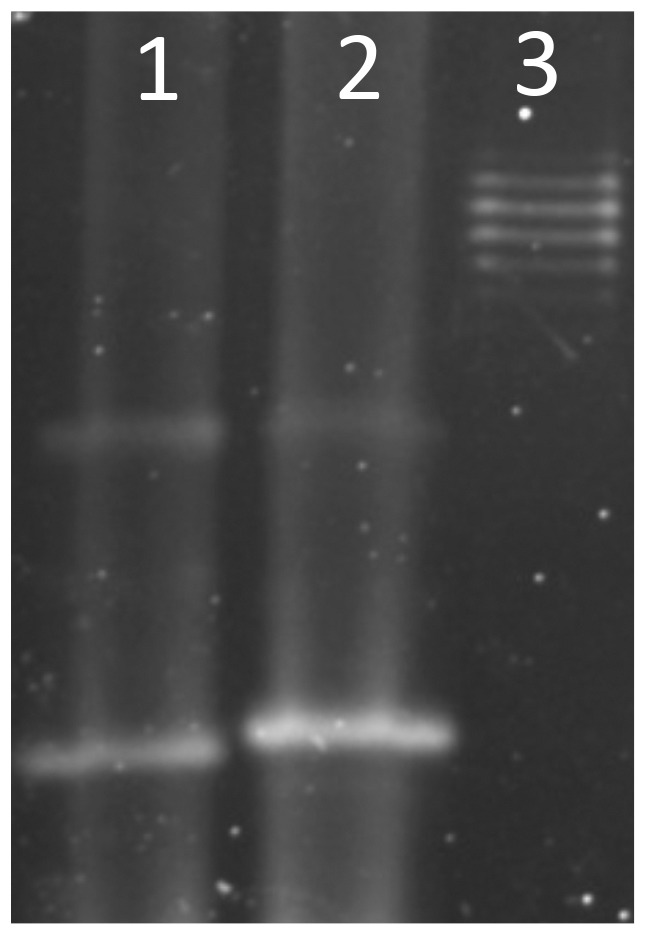
Lipooligosaccharide (LOS) profiles of *H. parasuis* strains Nagasaki and D74. LOS was extracted from *H. parasuis* Nagasaki (Lane 1) and D74 (Lane 2) and evaluated by electrophoresis and stained as described in **Materials and Methods** **.** LPS extracted from *E. coli* was included as a control (Lane 3).

Both Nagasaki and D74 genome sequences contain functional lipooligosaccharide (LOS) biosynthesis pathways, however several differences in the genes encoding glycosyltranferases and glucosyltransferases predicted to function in LOS biosynthesis were identified. LOS extraction and separation using polyacrylamide gel electrophoresis was performed on strains Nagasaki and D74 and although the same banding pattern was seen there were slight differences in the migration of the bands indicating possible structural alterations in the surface polysaccharides of these two strains (Figure 5).

Genes present exclusively in the genome sequences of either Nagasaki or D74, or divergent between the two, are indicated in Table S3 in File S1. Many of these genes could not have a function assigned based on sequence homology and were annotated only as hypothetical proteins. Because the D74 genome is not fully closed, some genes that were found only in the Nagasaki genome sequence could potentially lie in gapped regions of D74. Therefore, we narrowed our search for Nagasaki-specific genes of interest to those found within otherwise colinear regions of uninterrupted Nagasaki and D74 contigs. Among this group is the putative *vapD* (virulence-associated protein D) gene (HPSNAG_1862), also found in SH0165 (HAPS_1887), identified as contributing to epithelial cell invasion in *H. influenzae* [54]. An adjacent gene in Nagasaki, annotated as a hypothetical protein, is similarly missing in D74. A Nagasaki gene encoding a putative ABC transporter family protein is one of four contiguous ORFs (HPSNAG_0219-0222), also present in SH0165 that are deleted from this otherwise colinear region of the D74 genome and not found elsewhere. Zhou et al. identified a potential vaccine immunogen from SH0165 on the basis of its ability to induce bactericidal antibodies [53]. They reported the locus tag of the corresponding gene to be HPS_06257. However, no such locus tag exists in the SH0165 genome. When we queried the genome sequence with the primers reportedly used to amplify the gene we identified the locus as HPS_0911. This ORF is predicted to encode an outer membrane protein with a molecular mass of ∼28 Kd that contains a lipoprotein GNA1870 domain at its C-terminal end, in agreement with the characteristics previously ascribed to HPS_06257. A homolog in Nagasaki (HPSNAG_1786) with 99.6% amino acid identity to the SH0165 protein is absent from the D74 genome, with two tRNA genes at its expected location. Colinear sequences containing several contiguous ORFs predicted to encode genes related to iron acquisition or transport found in both Nagasaki (HPSNAG_0062-0068) and D74 (HPSD74_0040-0044) were noted to have regions of significant divergence. An ORF encoding the SH0165 putative adhesin AidA [31] is present in both the Nagasaki (HPSNAG_0727) and D74 (HPSD74_0870) genomes, though their predicted amino acid sequences are only ∼69% identical. Relative to the Nagasaki AidA protein, D74 has an insertion of 9 amino acids and another of 29 amino acids as well as several smaller insertions and many substitutions. As compared to AidA of SH0165, the Nagasaki protein is less divergent than that of D74 (80.5% versus 64.8% amino acid identity, respectively). Nonetheless, an autotransporter domain (PF03797.14) at the C-terminus is highly conserved in all. The variation identified among these and other gene sets documented in Table S3 in File S1 likely contributes to the increased virulence and disease severity exhibited by strain Nagasaki relative to strain D74. The significance of these differences can now be tested by genetic approaches and comparative genomics will continue to be valuable to understand the pathobiology of *H. parasuis.*

## Supporting Information

File S1 **This file contains Table S1, Table S2, and Table S3.** Table S1. *H. parasuis* genome assembly statistics summary. Table S2. Similarity of draft genome CDS sequences to *H. parasuis* SH0165 metabolic genes. Table S3. Genome comparison of strains Nagasaki and D74 listing individual coding sequences present in only one of the two strains or divergent between the two.
(XLSX)

## References

[1] Vahle JL, Haynes JS, Andrews JJ (1995) Experimental reproduction of *Haemophilus parasuis* infection in swine: clinical, bacteriological, and morphologic findings. J Vet Diagn Invest 7: 476–80. 8580168 10.1177/104063879500700409

[2] Vahle JL, Haynes JS, Andrews JJ (1997) Interaction of *Haemophilus parasuis* with nasal and tracheal mucosa following intranasal inoculation of cesarean derived colostrum deprived (CDCD) swine. Can J Vet Res 61: 200–206. 9243000 PMC1189404

[3] Riley MG, Russell EG, Callinan RB (1977) *Haemophilus parasuis* infection in swine. J Am Vet Med Assoc 171: 649–651. 914694

[4] Little TW (1970) *Haemophilus* infection in pigs. Vet Rec 87: 399–402. 5531588 10.1136/vr.87.14.399

[5] Brockmeier SL (2004) Prior infection with *Bordetella bronchiseptica* increases nasal colonization by *Haemophilus parasuis* in swine. Vet Microbiol 99: 75–78. 15019114 10.1016/j.vetmic.2003.08.013

[6] Holtkamp D, Rotto H, Garcia R (2007) Swine News Newsletter 30: 4.

[7] Solano-Aguilar GI, Pijoan C, Rapp-Gabrielson V, Collins J, Carvalho LF, et al (1999) Protective role of maternal antibodies against *Haemophilus parasuis* infection. Am J Vet Res 60: 81–87. 9918152

[8] Oliveira S, Batista L, Torremorell M, Pijoan C (2001) Experimental colonization of piglets and gilts with systemic strains of *Haemophilus parasuis* and *Streptococcus suis* to prevent disease. Can J Vet Res 65: 161–167. 11480521 PMC1189670

[9] Oliveira S, Galina L, Blanco I, Canals A, Pijoan C (2003) Naturally-farrowed, artificially reared pigs as an alternative model for experimental infection by *Haemophilus parasuis* . Can J Vet Res 67: 146–150. 12760482 PMC227044

[10] Blanco I, Galina-Pantoja L, Oliveira S, Pijoan C, Sanchez C, et al (2004) Comparison between *Haemophilus parasuis* infection in colostrum-deprived and sow-reared piglets. Vet Microbiol 103: 21–27. 15381262 10.1016/j.vetmic.2004.06.011

[11] Kielstein P, Rapp-Gabrielson VJ (1992) Designation of 15 serovars of *Haemophilus parasuis* on the basis of immunodiffusion using heat-stable antigen extracts. J Clin Microbiol 30: 862–865. 1572971 10.1128/jcm.30.4.862-865.1992 PMC265175

[12] Rapp-Gabrielson VJ, Gabrielson DA (1992) Prevalence of *Haemophilus parasuis* serovars among isolates from swine. Am J Vet Res 53: 659–664. 1524289

[13] Olvera A, Cerda-Cuellar M, Nofrarias M, Revilla E, Segales J, et al (2007) Dynamics of *Haemophilus parasuis* genotypes in a farm recovered from an outbreak of Glässer's disease. Vet Microbiol 123: 230–237. 17418506 10.1016/j.vetmic.2007.03.004

[14] Miniats OP, Smart NL, Ewert E (1991) Vaccination of gnotobiotic primary specific pathogen-free pigs against *Haemophilus parasuis* . Can J Vet Res 55: 33–36. 1832078 PMC1263410

[15] Miniats OP, Smart NL, Rosendal S (1991) Cross protection among *Haemophilus parasuis* strains in immunized gnotobiotic pigs. Can J Vet Res 55: 37–41. 1884282 PMC1263411

[16] Brockmeier SL, Loving CL, Mullins MA, Register KB, Nicholson TL, et al (2013) Virulence, transmission, and heterologous protection among four isolates of *Haemophilus parasuis* . Clin Vaccine Immunol 20: 1466–1472. 23885030 10.1128/CVI.00168-13 PMC3889593

[17] Fu S, Ou J, Zhang M, Xu J, Liu H, et al (2013) The live attenuated *Actinobacillus pleuropneumoniae* triple-deletion mutant ΔapxIC ΔapxIIC ΔapxIV-ORF1 strain, SLW05, immunizes pigs against lethal challenge with *Haemophilus parasuis* . Clin Vaccine Immunol 20: 134–139. 23220998 10.1128/CVI.00458-12 PMC3571286

[18] Cerda-Cuellar M, Aragon V (2008) Serum-resistance in *Haemophilus parasuis* is associated with systemic disease in swine. Vet J 175: 384–389. 17368943 10.1016/j.tvjl.2007.01.016

[19] Olvera A, Ballester M, Nofrarias M, Sibila M, Aragon V (2009) Differences in phagocytosis susceptibility in *Haemophilus parasuis* strains. Vet Res 40: 24–35. 19239855 10.1051/vetres/2009007 PMC2695031

[20] Pina S, Olvera A, Barcelo A, Bensaid A (2009) Trimeric autotransporters of *Haemophilus parasuis*: generation of an extensive passenger domain repertoire specific for pathogenic strains. J Bacteriol 191: 576–587. 19011035 10.1128/JB.00703-08 PMC2620822

[21] Costa-Hurtado M, Ballester M, Galofre-Mila N, Darji A, Aragon V (2012) VtaA8 and VtaA9 from *Haemophilus parasuis* delay phagocytosis by alveolar macrophages. Vet Res 43: 57–66. 22839779 10.1186/1297-9716-43-57 PMC3462726

[22] Zhang B, Feng S, Xu C, Zhou S, He Y, et al (2012) Serum resistance in *Haemophilus parasuis* SC096 strain requires outer membrane protein P2 expression. FEMS Microbiol Lett 326: 109–115. 22092746 10.1111/j.1574-6968.2011.02433.x

[23] Zhang B, He Y, Xu C, Xu L, Feng S, et al (2012) Cytolethal distending toxin (CDT) of the *Haemophilus parasuis* SC096 strain contributes to serum resistance and adherence to and invasion of PK-15 and PUVEC cells. Vet Microbiol 157: 237–242. 22221379 10.1016/j.vetmic.2011.12.002

[24] Yue M, Yang F, Yang J, Bei W, Cai X, et al (2009) Complete genome sequence of *Haemophilus parasuis* SH0165. J Bacteriol 191: 1359–1360. 19074396 10.1128/JB.01682-08 PMC2632009

[25] Blackall PJ, Turni C (2013) Understanding the virulence of *Haemophilus parasuis* . Vet J 198: 549–550. 24268480 10.1016/j.tvjl.2013.09.070

[26] Amano H, Shibata M, Kajio N, Morozumi T (1994) Pathologic observations of pigs intranasally inoculated with serovar 1, 4 and 5 of *Haemophilus parasuis* using immunoperoxidase method. J Vet Med Sci 56: 639–644. 7999883 10.1292/jvms.56.639

[27] Amano H, Shibata M, Kajio N, Morozumi T (1996) Pathogenicity of *Haemophilus parasuis* serovars 4 and 5 in contact-exposed pigs. J Vet Med Sci 58: 559–561. 8811627 10.1292/jvms.58.559

[28] Frandoloso R, Martínez S, Rodríguez-Ferri EF, García-Iglesias MJ, Pérez-Martínez C, et al (2011) Development and characterization of protective *Haemophilus parasuis* subunit vaccines based on native proteins with affinity to porcine transferrin and comparison with other subunit and commercial vaccines. Clin Vaccine Immunol 18: 50–58. 20926701 10.1128/CVI.00314-10 PMC3019774

[29] Olvera A, Pina S, Pérez-Simó M, Aragón V, Segalés J, et al (2011) Immunogenicity and protection against *Haemophilus parasuis* infection after vaccination with recombinant virulence associated trimeric autotransporters (VtaA). Vaccine 29: 2797–2802. 21320547 10.1016/j.vaccine.2011.01.105

[30] Rapp-Gabrielson VJ, Gabrielson DA, Schamber GJ (1992) Comparative virulence of *Haemophilus parasuis* serovars 1 to 7 in guinea pigs. Am J Vet Res 53: 987–994. 1626790

[31] Xu Z, Yue M, Zhou R, Jin Q, Fan Y, et al (2011) Genomic characterization of *Haemophilus parasuis* SH0165, a highly virulent strain of serovar 5 prevalent in China. PLoS One 6: e19631. 21611187 10.1371/journal.pone.0019631 PMC3096633

[32] Mullins MA, Register KB, Bayles DO, Dyer DW, Kuehn JS, et al (2011) Genome sequence of *Haemophilus parasuis* strain 29755. Stand Genomics 5: 61–68. 10.4056/sigs.2245029 PMC3236040 22180811

[33] Kielstein P, Rosner H, Muller W (1991) Typing of heat-stable soluble *Haemophilus parasuis* antigen by means of agar gel precipitation and the dot-blot procedure. J Vet Med B 38: 315–320. 10.1111/j.1439-0450.1991.tb00877.x 1832258

[34] Mullins MA, Register KB, Brunelle BW, Aragon V, Galofre-Mila N, et al (2013) A curated public database for multilocus sequence typing (MLST) and analysis of *Haemophilus parasuis* based on an optimized typing scheme. Vet Microbiol 162: 899–906. 23218953 10.1016/j.vetmic.2012.11.019

[35] Quail MA, Kozarewa I, Smith F, Scally A, Stephens PJ, et al (2008) A large genome center's improvements to the Illumina sequencing system. Nat Methods 5: 1005–1010. 19034268 10.1038/nmeth.1270 PMC2610436

[36] Kuehn JS, Register KB, Phillips GJ (2013) Draft Genome Sequences for 10 Isolates of the Swine Pathogen *Haemophilus parasuis* . Genome Announc 1: e00739–13 doi: 10.1128/genomeA.00739-13 24051319 10.1128/genomeA.00739-13 PMC3778202

[37] Shao Y, He X, Harrison EM, Tai C, Ou HY, et al (2010) mGenomeSubtractor: a web-based tool for parallel in silico subtractive hybridization analysis of multiple bacterial genomes. Nucleic Acids Res 38: W194–200. 20435682 10.1093/nar/gkq326 PMC2896100

[38] Chevreux B, Wetter T, Suhai S (1999) Genome sequence assembly using trace signals and additional sequence information. Computer Science and Biology: Proceedings of the German Conference on Bioinformatics (GCB) 99, pp. 45–56.

[39] Darling AC, Mau B, Blattner FR, Perna NT (2004) Mauve: multiple alignment of conserved genomic sequence with rearrangements. Genome Res 14: 1394–1403. 15231754 10.1101/gr.2289704 PMC442156

[40] Darling AE, Mau B, Perna NT (2010) progressiveMauve: multiple genome alignment with gene gain, loss and rearrangement. PLoS One 5: e11147. 20593022 10.1371/journal.pone.0011147 PMC2892488

[41] Edgar RC (2004) MUSCLE: multiple sequence alignment with high accuracy and high throughput. Nucleic Acids Res 32: 1792–1797. 15034147 10.1093/nar/gkh340 PMC390337

[42] Larkin MA, Blackshields G, Brown NP, Chenna R, McGettigan PA, et al (2007) Clustal W and Clustal X version 2.0. Bioinformatics 23: 2947–2948. 17846036 10.1093/bioinformatics/btm404

[43] McWilliam H, Valentin F, Goujon M, Li W, Narayanasamy M, et al (2009) Web services at the European Bioinformatics Institute-2009. Nucleic Acids Res 37: W6–10. 19435877 10.1093/nar/gkp302 PMC2703973

[44] Aziz RK, Bartels D, Best AA, DeJongh M, Disz T, et al (2008) The RAST Server: Rapid Annotations using Subsystems Technology. BMC Genomics 9. 10.1186/1471-2164-9-75 PMC2265698 18261238

[45] Stothard P, Wishart DS (2005) Circular genome visualization and exploration using CGView. Bioinformatics 21: 537–539. 15479716 10.1093/bioinformatics/bti054

[46] Eddy SR (2011) Accelerated Profile HMM Searches. PLoS Comput Biol 7: e1002195. 22039361 10.1371/journal.pcbi.1002195 PMC3197634

[47] Yoon SH, Park YK, Lee S, Choi D, Oh TK, et al (2007) Towards pathogenomics: a web-based resource for pathogenicity islands. Nucleic Acids Research 35: D395–D400. 17090594 10.1093/nar/gkl790 PMC1669727

[48] Apicella MA (2008) Bacterial Pathogenesis: Methods in Molecular Biology, volume 431, pp 3–13. Isolation and Characterization of Lipopolysaccharides. Available: http://link.springer.com/protocol/10.1007/978-1-60327-032-8_1 10.1007/978-1-60327-032-8_1 18287743

[49] Costa-Hurtado M, Olvera A, Martinez-Moliner V, Galorfre-Mila N, Martinez P, et al (2013) Changes in macrophage phenotype after infection of pigs with *Haemophilus parasuis* strains with different levels of virulence. Infect Immun 81: 2327–2333. 23589574 10.1128/IAI.00056-13 PMC3697589

[50] Howell KJ, Weinert LS, Luan SL, Peters SE, Chaudhuri RR, et al (2013) Gene content and diversity of the loci encoding biosynthesis of capsular polysaccharides of the 15 serovar reference strains of *Haemophilus parasuis* . J Bacteriol 195: 4264–4273. 23873912 10.1128/JB.00471-13 PMC3754760

[51] Li Y, Kwok AH, Jiang J, Zou Y, Zheng F, et al (2013) Complete genome analysis of a *Haemophilus parasuis* serovar 12 strain from China. PLoS One 8: e68350. 24023711 10.1371/journal.pone.0068350 PMC3759607

[52] Yuan F, Fu S, Hu J, Li J, Chang H, et al (2012) Evaluation of recombinant proteins of *Haemophilus parasuis* strain SH0165 as vaccine candidates in a mouse model. Res Vet Sci 93: 51–56. 21596404 10.1016/j.rvsc.2011.04.020

[53] Zhou M, Guo Y, Zhao J, Hu Q, Hu Y, et al (2009) Identification and characterization of novel immunogenic outer membrane proteins of *Haemophilus parasuis* serovar 5. Vaccine 27: 5271–5277. 19576561 10.1016/j.vaccine.2009.06.051

[54] Daines DA, Jarisch J, Smith AL (2004) Identification and characterization of a nontypeable *Haemophilus influenzae* putative toxin-antitoxin locus. BMC Microbiology 4: 30. 15274747 10.1186/1471-2180-4-30 PMC503385

